# Chronic dietary supplementation with kynurenic acid, a neuroactive metabolite of tryptophan, decreased body weight without negative influence on densitometry and mandibular bone biomechanical endurance in young rats

**DOI:** 10.1371/journal.pone.0226205

**Published:** 2019-12-06

**Authors:** Ewa Tomaszewska, Siemowit Muszyński, Damian Kuc, Piotr Dobrowolski, Krzysztof Lamorski, Katarzyna Smolińska, Janine Donaldson, Izabela Świetlicka, Maria Mielnik-Błaszczak, Piotr Paluszkiewicz, Jolanta Parada-Turska

**Affiliations:** 1 Department of Animal Physiology, University of Life Sciences in Lublin, Lublin, Poland; 2 Department of Biophysics, University of Life Sciences in Lublin, Lublin, Poland; 3 Department of Developmental Age Stomatology, Chair of Developmental Age Stomatology, Medical University of Lublin, Lublin, Poland; 4 Department of Comparative Anatomy and Anthropology, Maria Curie-Sklodowska University, Lublin, Poland; 5 Bohdan Dobrzański Institute of Agrophysics of the Polish Academy of Sciences, Lublin, Poland; 6 Department of Surgery and Surgical Nursing, Medical University of Lublin, Lublin, Poland; 7 School of Physiology, Faculty of Health Sciences, University of the Witwatersrand, Parktown, South Africa; 8 Department of General, Oncological and Metabolic Surgery, Institute of Haematology and Transfusion Medicine, Warszawa, Poland; 9 Department of Rheumatology and Connective Tissue Diseases, Medical University of Lublin, Lublin, Poland; Macquarie University, AUSTRALIA

## Abstract

Kynurenic acid (KYNA) is a neuroactive metabolite of tryptophan. KYNA naturally occurs in breast milk and its content increases with lactation, indicating the role of neonatal nutrition in general growth with long-term health effects. KYNA is also an antagonist of ionotropic glutamate receptors expressed in bone cells. The aim of this study was to establish the effects of chronic KYNA supplementation on bone homeostasis in young rats, using mandible as a model bone. Female and male newborn Wistar rats were divided into control and KYNA-administered groups until 60 days of age (25x10^1^ mg/L or 25x10^2^ mg/L in drinking water). Hemimandibles were subjected to densitometry, computed tomography analysis and mechanical testing. Rats supplemented with KYNA at both doses showed a decrease in body weight. There were no effects of KYNA administration and mandible histomorphometry. In males, a significant quadratic effect (P < 0.001) was observed in the densitometry of the hemimandible, where BMD increased in the group supplemented with 2.5x10^1^ mg/L of KYNA. Analysis of mechanical tests data showed that when fracture forces were corrected for bone geometry and rats body weight the improvement of bone material properties was observed in male and female rats supplemented with lower dose of KYNA. This study showed that chronic supplementation with KYNA may limit weight gain in the young, without adversely affecting the development of the skeleton.

## Introduction

Metabolites of tryptophan, an essential amino acid, have been the subject of intense research activity over the past two decades, their important roles in neuronal and immune function have been discovered [1]. Tryptophan is transformed either to tryptamine and serotonin or through kynurenine pathway leading to the production of kynurenic acid (KYNA), and through intermediate metabolites as 3-hydroxykynurenine, 3-hydroxyanthranilic acid to picolinic acid or quinolinic acid and NAD+ [1, 2].

KYNA occurs naturally in living organisms in the brain, saliva, cerebrospinal fluid, blood serum, liver, intestines, kidneys, cardiac muscle and endothelium [2–8]. KYNA also is present in breast milk and its content increases with lactation indicating the role of neonatal nutrition in general growth with long-term health effects [9]. Furthermore, exogenous KYNA is an ingredient of food including commercial baby formulas and honey [9, 10]. The deficiency in dietary KYNA could result in dysfunction of adipose tissue, which plays important endocrine functions in regulation of energy homeostasis, insulin sensitivity, lipid and carbohydrate metabolism and it could result in overweight. There is a clear correlation between plasma levels of tryptophan and Body Mass Index (BMI) [11].

KYNA is also an antagonist of ionotropic glutamate receptors, including the N-methyl-D-aspartate (NMDA) receptor [12, 13]. Glutamate receptors are located predominantly in the central nervous system (CNS); however they are present on the surface of cells in peripheral organs and tissues, as well as in endocrine cells [14, 15]. Importantly, glutamate receptors, transporters and proteins that regulate glutamate release are expressed in osteoblasts, osteocytes and osteoclasts, and influence the activities of these cells [16]. Bone cells could express all the molecular machinery required for glutamate signaling in the CNS [17]. Since glutamate receptors are expressed on osteoclast cells, it is possible that KYNA could influence bone remodeling [18]. Furthermore, previous study has demonstrated that activation of the kynurenine pathway is associated with osteoblastogenesis, which has been implicated in the occurrence of bone diseases [19].

Bone tissue homeostasis fluctuates with age and depends on hormonal and nutritional modifications [20, 21]. The quality of food consumed plays an important role in general development including that of bone. The beneficial impact of specific compounds in food is evident. Nutrient deficiencies or excess, in conjunction with coexisting physiological processes, could have hyper-additive effects on biological systems, thus possibly resulting in increased harmful or beneficial effects [20, 22].

As recently suggested, there could be a difference in the peripheral and central action of kynurenine on bone metabolism [23]. Beneficial peripheral effects of KYNA including anti-atherosclerosis, anti-ulcer, anti-inflammatory and anti-migraine actions, have previously been documented [24–26]. The role of KYNA in peripheral tissues including neonatal period has not yet been established. Therefore, the aim of this study was to establish the effects of chronic KYNA administration in drinking water on bone development in neonatal and early postnatal time on the rat model.

## Materials and methods

The rats were housed in the Center of Experimental Medicine in the Medical University of Lublin, Poland. All experimental procedures were carried out in accordance with the guidelines of the European Parliament and of the Council of 22 September 2010, on the protection of animals used for scientific purposes (2010/63/EU) and approved by the Local Ethics Committee for Animal Experimentation in Lublin, Poland (37/2017).

### Animal, breeding and experimental design

Seventy-two newborn Wistar rats, were used in the current study. The dams with their offspring were kept under standard laboratory conditions (a 12-h light-dark cycle, temperature of 21±1°C, humidity 55±5%) in colony cages, and fed standard laboratory rodents diet ad libitum. After delivery the rats were kept with their mothers from postnatal day, until weaning at the age of 21. After weaning, pups were maintained to the age of 60 days.

The dams with their litter were randomly divided into three groups. Control rats had free access to fresh tap water without KYNA. In two other groups, KYNA (kynurenic acid, Sigma–Aldrich, St. Louis, MO, USA) was administered in the drinking water in concentrations of 25x10^1^ mg/L (approx. 2.5 mg/kg body weight/day) or 25x10^2^ mg/L (approx. 25 mg/kg body weight/day) to exclude KYNA accumulation and potential toxicity. Equal number of male (n = 12) and females (n = 12) was in each group.

Rats were weighed six times, starting from the 21^st^ day, up to the age of 60 days when were sacrificed by decapitation. The right hemimandible from each rat was collected and wrapped in gauze soaked in isotonic PhS and frozen at -25ºC until further analysis. The incisor was not removed during all subsequent experimental procedures because removal could potentially cause fracture, damaging the trabecular bone and possibly altering hemimandible biomechanical endurance [27].

### Bone mineral density and computed tomography analysis of hemimandibles

Bone mineral density (BMD) was assessed using the XR 43 Norland DXA densitometer (Fort Atkinson, WI, USA). X-ray computed tomography analysis was conducted using a Nanotom 180S device (GE Sensing & Inspection Technologies GmbH, Wunstorf, Germany) with a rotation step of 0.3 deg and the scan resolution 20 μm. The parameters of the XRT acquisition were: X-ray source voltage 140 kV, X-ray source current 250 μA, and a 0.3 mm Cu filter.

**Fig 1:**
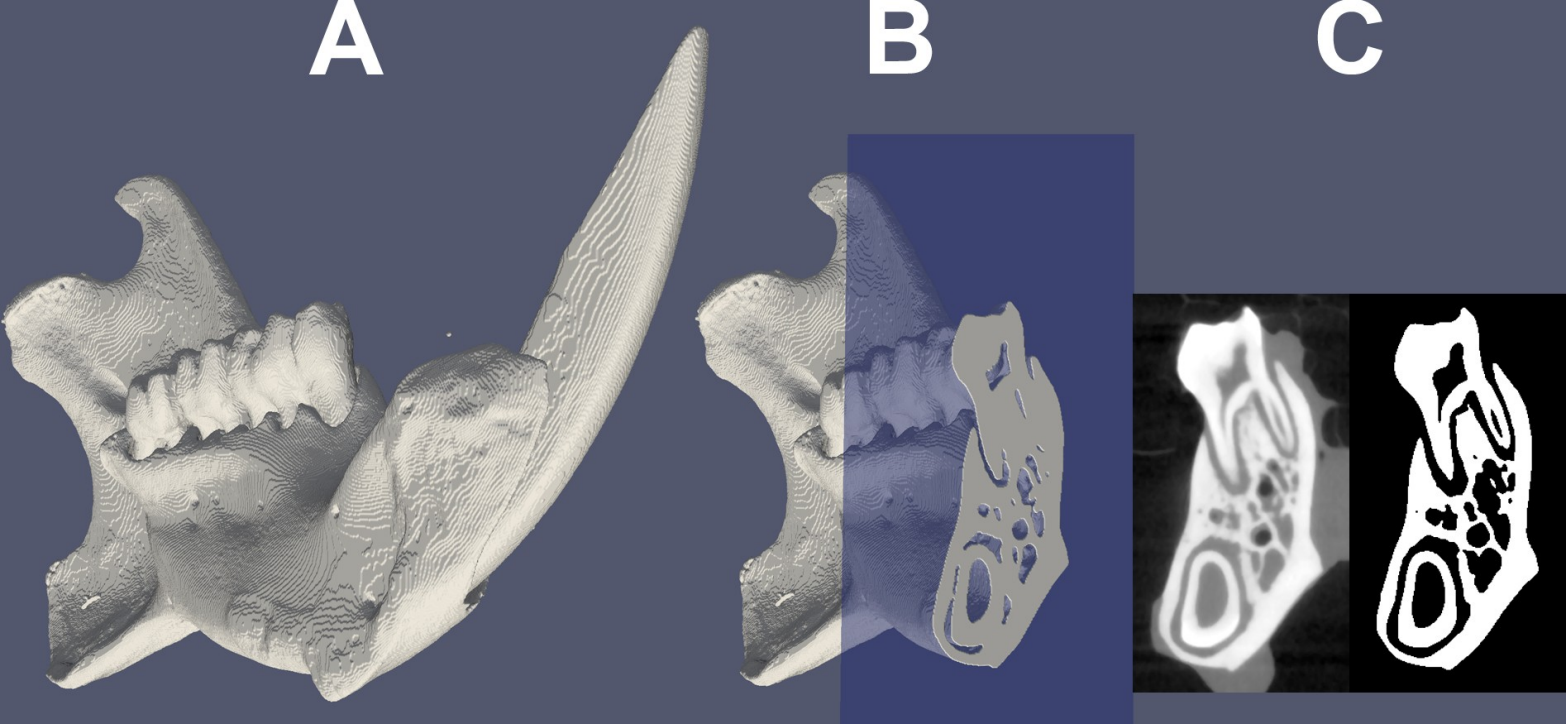
(A) 3D reconstruction image of a single hemimandible; (B) Selection of 2D cross section; (C) Processing of the obtained images: filtering, thresholding (left) and final conversion to 8 bit format (right).

**Fig 2:**
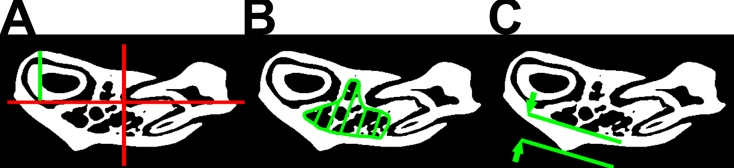
The analysis of thresholded images. (A) The position of neutral axis (red) of centroid and maximal perpendicular distance (green) from the neutral cranial-caudal axis. Designation of region of interest (ROI) for (B) the trabecular bone morphometry and (C) cortical bone thickness. B, C adapted from [29].

The 3D reconstruction was done using DatosX 2.0 software (GE Sensing & Inspection Technologies GmbH, Wunstorf, Germany) and 16 bit grey-level 3D images were generated (Fig 1A). Image analysis was performed using VG Studio Max 2.0 (Volume Graphics GmbH, Heidelberg, Germany), Fiji (NIH, Bethesda, MA, USA) and Avizo 9 (FEI, Hillsboro, Oregon, USA) software. The 2D cross section image were extracted in coronal plane in the region below the first molar (Fig 1B). The cross sections obtained were then further analysed as detailed below. Thresholding was done using IsoData algorithm [28] with thorough inspection of the thresholded images (Fig 1C). The position of the horizontal neutral axis of centroid and cross-sectional moment of inertia about the neutral cranial-caudal axis for transformed images were determined using the appropriate tool in Fiji’s BoneJ plugin. Then the maximal perpendicular distance from the neutral C-C axis was measured (Fig 2A). The trabecular bone morphometry was measured using the pixel count on the transformed images in manually designated ROIs using Fiji’s software (Fig 2B). Cortical bone thickness was measured as the length of the masseter’s ridge, perpendicular to the inner cortical surface (Fig 2C) [29].

### Hemimandible biomechanical testing

**Fig 3:**
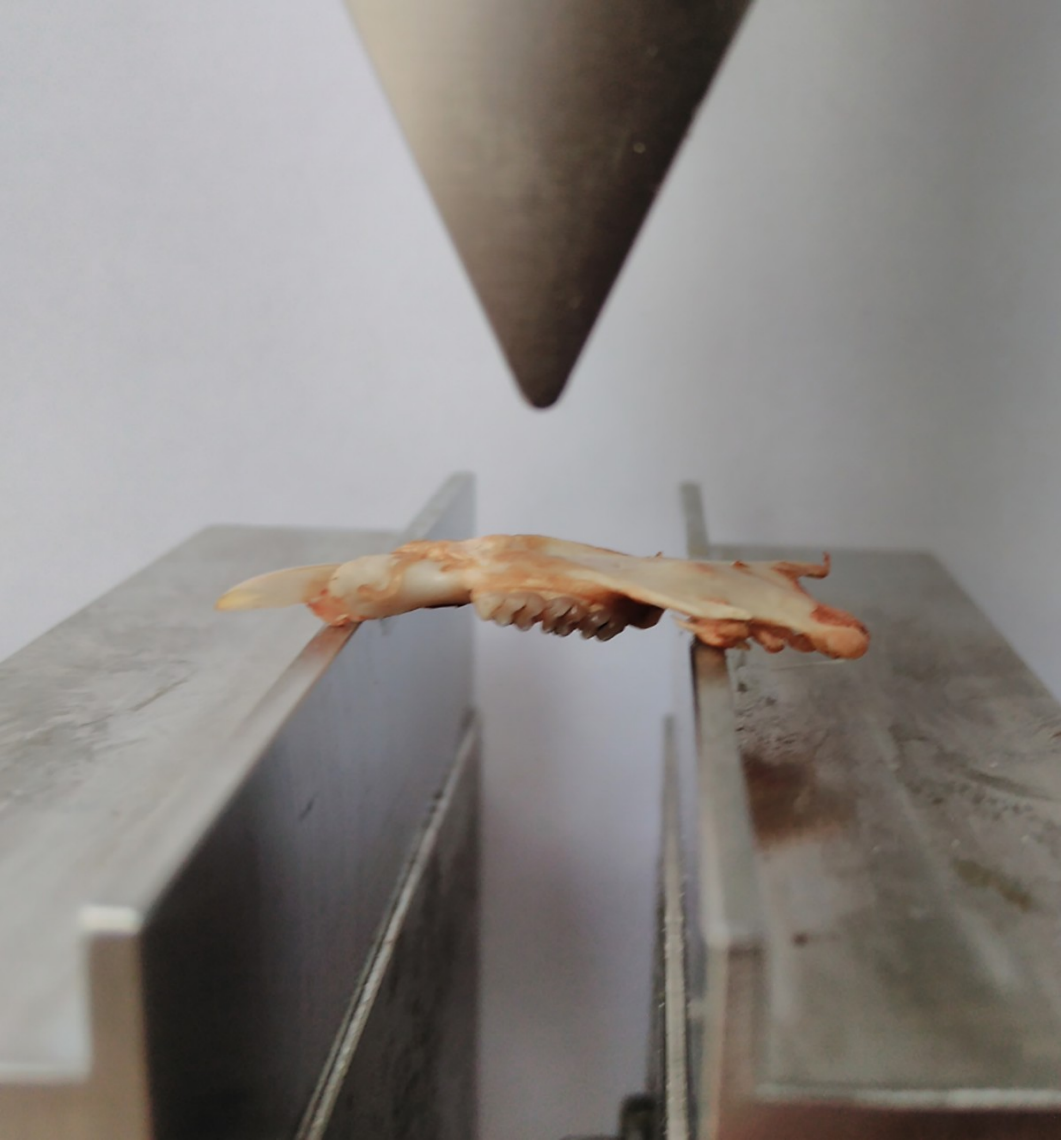
Positioning of the hemimandible for three-point bending test.

Hemimandible mechanical strength was determined using the three-point bending test on a universal testing machine (Zwick Z010, Zwick/Roell, Ulm, Germany). The bone was placed on the custom-made supports (14 mm span) with the buccal side upward (Fig 3). The loading point was aligned at the first molar midpoint (Jiang, et al., 2008). The load was applied at a constant rate of 5 mm/min until fracture [30].

Hemimandible structural properties were determined from the force-displacement curves using Origin 2016 software (OriginLab, Northampton, MA, USA). The yield load was determined as maximal force under elastic (reversible) deformation and the ultimate load as the force causing fracture. Stiffness was measured as the slope of the elastic part of load-displacement curve; elastic energy, as the energy absorbed by bone in pre-yield, elastic region, and work to fracture, as a total work required to break or total energy absorbed by hemimandible until fracture [31]. The values of yield load and ultimate load were also normalized to rat weight, and expressed as relative yield load and ultimate load, respectively.

Whole-bone material properties (intrinsic properties of bone tissue) were calculated on the basis of appropriate engineering equations, using strength data obtained in three-point bending test and determined cross-sectional moment of inertia, which reflects the spatial distribution of bone tissue and describes the geometric contribution of the bone to resisting bending [31]. Young modulus of elasticity describes bending resistance of the hemimandible, yield strain and ultimate strain describe the relative deformation which occur when the specific load is applied, elastic stress reflects the elastic strength and the ultimate stress is equal to the maximum stress hemimandible can withstand in bending before fracture [31].

### Statistical analysis

All data were statistically analyzed using the general linear model procedure of the Statistica program (TIBCO Software Inc., Palo Alto, CA, USA). Polynomial contrasts were used to determine linear and quadratic effects of increasing KYNA levels on all measurements. A level of P < 0.05 was used to determine statistical significance.

## Results

**Fig 4:**
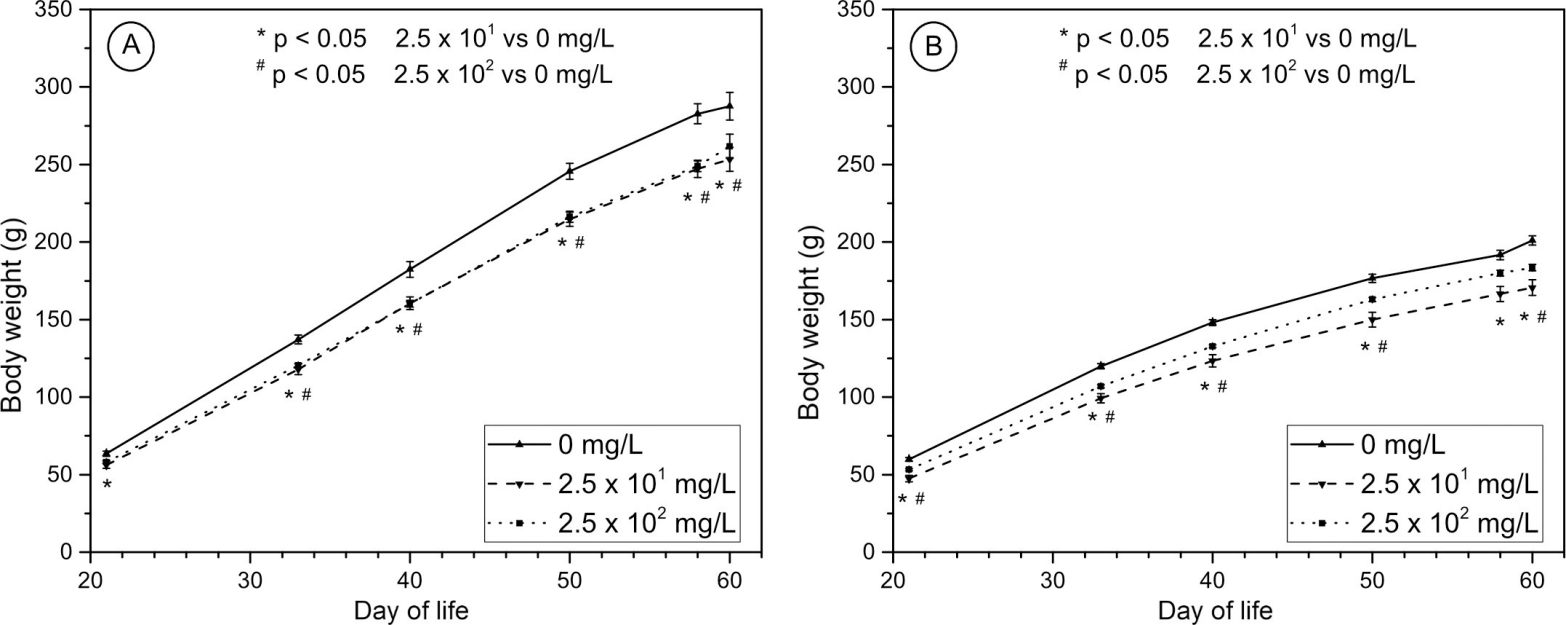
**Effect of KYNA administration in drinking water on body weight gains of male (A) and female (B) rats from the weaning at 21**^**st**^ **day, up to the age of 60 days.** Data are presented as the mean±SE (n = 12). Statistical analysis was performed using one-way ANOVA; p < 0.05.

Male and female rats supplemented with KYNA showed a decrease in body weight in all periods, when rats’ body weight was recorded (Fig 4). At the end of the experiment both linear (P < 0.05 and P < 0.01, for male and female, respectively) and quadratic (P < 0.05 and P < 0.001, for male and female, respectively) effect of KYNA supplementation was observed (Tables 1 and 2).

**Table 1:** Body weight and mandible mechanical properties of male, control and kynurenic acid-treated, Wistar rats.

Dependent variable	KYNA ^1^, mg/L			*P*-value	
	0	2.5 x 10^1^	2.5 x 10^2^	Linear	Quadratic
Body weight, g	287±9	253±8	262±8	0.032	0.040
Yield load, N	84.1±6.5	77.8±5.3	75.8±5.5	0.318	0.763
Ultimate load, N	132±6	145±6	133±5	0.918	0.084
Stiffness, N/mm	381±22	410±23	407±20	0.393	0.556
Elastic energy, mJ	13.6±2.5	8.11±0.86	7.98±1.16	0.024	0.201
Work to fracture, mJ	54.3±9.2	48.6±5.5	42.4±6.3	0.247	0.977
Relative yield load, N/g	0.292±0.020	0.310±0.022	0.290±0.019	0.933	0.453
Relative ultimate load, N/g	0.491±0.016	0.576±0.022	0.512±0.020	0.073	<0.001
Young modulus, MPa	1077±75	1322±87	1159±59	0.442	0.033
Yield strain, %	3.98±0.57	3.03±0.22	3.15±0.31	0.147	0.274
Elastic stress, MPa	39.3±3.2	38.9±2.8	35.5±2.8	0.360	0.670
Ultimate strain, %	11.8±1.8	9.66±1.03	8.63±0.93	0.095	0.725
Ultimate stress, MPa	60.5±2.3	72.7±3.2	60.7±2.1	0.973	<0.001

^1^ Data are presented as the mean±SE (n = 12).

**Table 2:** Body weight and mandible mechanical properties of female, control and kynurenic acid-treated, Wistar rats.

Dependent variable	KYNA ^1^, mg/L			*P*-value	
	0	2.5 x 10^1^	2.5 x 10^2^	Linear	Quadratic
Body weight, g	201±3	171±5	183±2	0.002	<0.001
Yield load, N	87.9±5.8	89.9±4.5	73.5±2.8	0.043	0.117
Ultimate load, N	137±6	142±4	139±4	0.954	0.543
Stiffness, N/mm	382±19	396±19	410±13	0.276	0.991
Elastic energy, mJ	11.0±1.2	10.2±1.2	7.19±0.37	0.065	0.512
Work to fracture, mJ	34.4±2.4	45.9±3.2	51.4±3.7	<0.001	0.427
Relative yield load, N/g	0.441±0.033	0.538±0.040	0.402±0.016	0.415	0.006
Relative ultimate load, N/g	0.689±0.026	0.838±0.023	0.761±0.023	0.048	<0.001
Young modulus, MPa	1238±85	1331±89	1332±58	0.418	0.632
Yield strain, %	3.86±0.42	3.75±0.32	2.99±0.11	0.076	0.416
Elastic stress, MPa	45.0±3.2	50.0±2.9	39.6±1.8	0.197	0.021
Ultimate strain, %	8.12±0.75	9.18±0.60	10.16±0.40	0.033	0.974
Ultimate stress, MPa	69.1±2.2	79.9±2.9	73.8±2.8	0.234	0.026

^1^ Data are presented as the mean±SE (n = 12).

While KYNA supplementation did not alter ultimate load in males, a significant quadratic effect (P < 0.001) was observed when the load was normalized to rat’s body weight (relative ultimate load), where a higher value was noted in the group supplemented with 2.5x10^1^ mg/L of KYNA compared to the control group. The relative ultimate load obtained in the group supplemented with the higher dose of KYNA was not different from that noted in the control group. Similar effects were observed for Young modulus and ultimate stress (quadratic, P < 0.05 and P < 0.001, respectively; Table 1). Elastic energy in male rats decreased with increasing dose of KYNA (linear, P < 0.05). There were no significant differences between groups in any of the other mandible mechanical properties evaluated.

Female rats that drank water enriched with KYNA showed a decrease in mandible yield load, with increasing doses of KYNA (linear, P < 0.05, Table 2). Moreover, significant linear effects were observed in work to fracture and ultimate strain in female rats (P < 0.001 and P < 0.05, respectively), with the highest values noted in the group supplemented with 2.5x10^2^ mg/L of KYNA. However, a significant quadratic effect (P < 0.01) was observed in relative yield load, where the highest value was noted in the group supplemented with 2.5x10^1^ mg/L of KYNA. KYNA administration also increased relative ultimate load (linear and quadratic, P < 0.05 and P < 0.001, respectively), with the highest value observed in the group supplemented with 2.5x10^1^ mg/L of KYNA. A significant quadratic effect (P < 0.05) was observed in both elastic stress and ultimate stress in female rats, with the highest values noted in the group supplemented with 2.5x10^1^ mg/L of KYNA, compared to the control group and those supplemented with 2.5x10^2^ mg/L of KYNA. Moreover, the elastic stress obtained in the group supplemented with the higher dose of KYNA was also significantly lower compared to that of the control group (Table 2). No other significant changes were observed.

In males, a significant quadratic effect (P < 0.001) was observed with regards to the densitometry, where BMD increased in the group supplemented with 2.5x10^1^ mg/L of KYNA compared to the control group and those supplemented with 2.5x10^2^ mg/L of KYNA. The BMD in the group supplemented with the higher dose of KYNA was not different from that noted in the control group (Table 3). An opposite quadratic effect (P < 0.001) was observed for cross-sectional moment of inertia, where a lower value was noted in the group supplemented with 2.5x10^1^ mg/L of KYNA compared to the control group and those supplemented with 2.5x10^2^ mg/L of KYNA.

**Table 3:** Densitometric parameters, trabecular bone morphometry and geometric parameters of the mandible of male, control and kynurenic acid-treated, Wistar rats.

Dependent variable	KYNA ^1^, mg/L			*P*-value	
	0	2.5 x 10^1^	2.5 x 10^2^	Linear	Quadratic
BMD, g/cm^2^	0.145±0.002	0.155±0.002	0.144±0.001	0.739	<0.001
BV/TV, %	33.5±2.3	34.0±2.5	30.5±1.4	0.334	0.446
Tb.Th mean, μm	179±9	170±7	165±6	0.213	0.859
Tb.Th max, μm	302±15	306±14	302±19	1.000	0.826
Tb.Sp mean, μm	553±28	540±27	573±38	0.643	0.552
Tb.Sp max, μm	1019±48	989±53	1097±76	0.372	0.361
Tb.N, 1/mm	1.87±0.08	1.97±0.06	1.85±0.04	0.793	0.179
Cortical.Th, μm	778±33	737±21	773±13	0.898	0.204
CSMI, mm^4^	7.60±0.28	6.89±0.24	7.56±0.27	0.900	0.041

^1^ Data are presented as the mean±SE (n = 12). BMD–bone mineral density, BV/TV–relative bone volume; Tb.Th–trabecular thickness; Tb.Sp–trabecular separation; Tb.N–trabecular number; Cortical.Th–cortical bone thickness; CSMI–cross-sectional moment of inertia.

**Fig 5:**
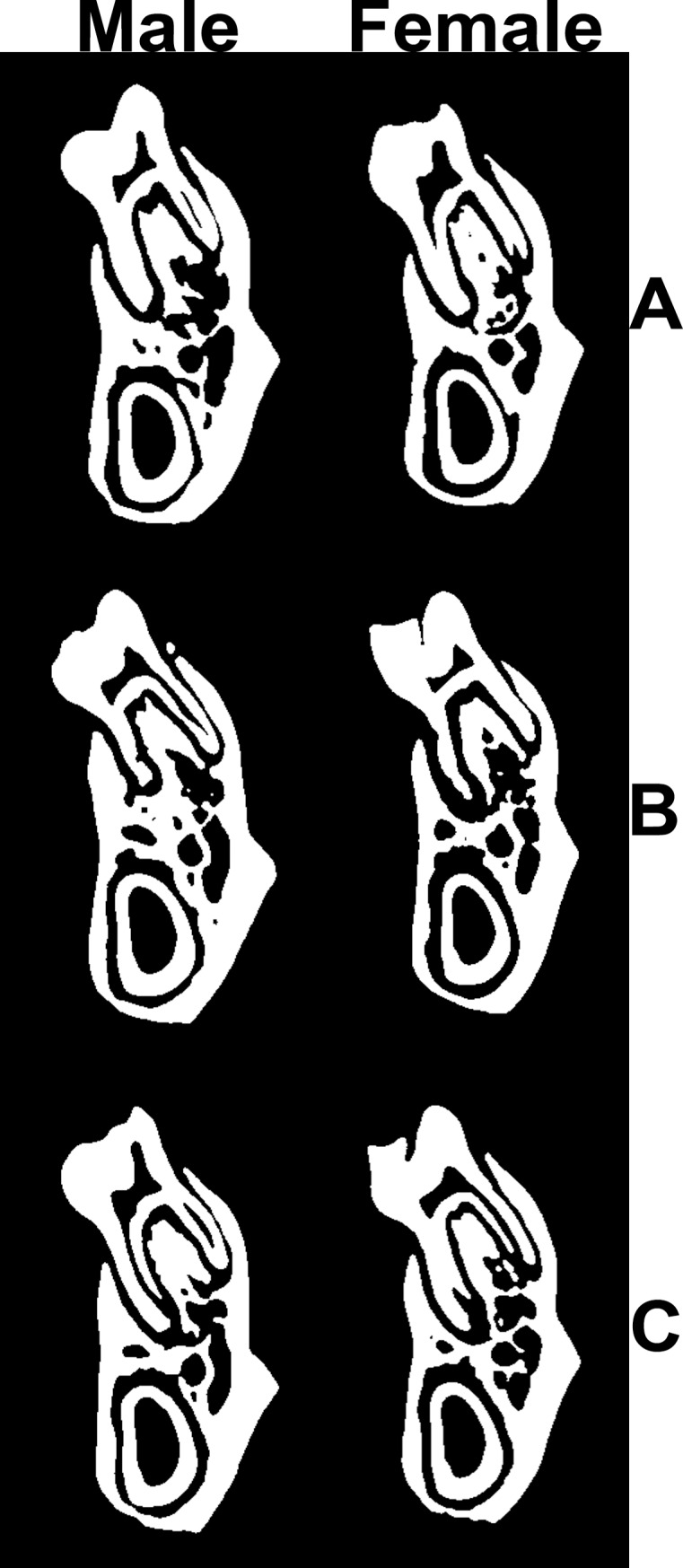
Cross-sectional images of representative hemimandibles of male (left) and female (right) rats from the control group (A) and from rats chronically supplemented with KYNA in concentrations of 25x10^1^ mg/L (B) or 25x10^2^ mg/L (C) in drinking water.

In female rats the lowest cross-sectional moment of inertia was observed in the group supplemented with the lower dose of KYNA (quadratic, P < 0.05, Table 4). No significant differences in trabecular bone morphology or cortical bone thickness were observed between groups, for both male and female rats (Tables 3 and 4; Fig 5).

**Table 4:** Densitometric parameters, trabecular bone morphometry and geometric parameters of the mandible of female, control and kynurenic acid-treated, Wistar rats.

Dependent variable	KYNA ^1^, mg/L			*P*-value	
	0	2.5 x 10^1^	2.5 x 10^2^	Linear	Quadratic
BMD, g/cm^2^	0.146±0.003	0.140±0.002	0.143±0.003	0.556	0.168
BV/TV, %	34.1±1.6	32.2±1.5	35.0±1.5	0.690	0.192
Tb.Th mean, μm	156±6	160±8	163±5	0.490	0.988
Tb.Th max, μm	295±17	280±13	295±12	0.983	0.370
Tb.Sp mean, μm	505±42	508±29	484±19	0.656	0.734
Tb.Sp max, μm	966±79	984±71	889±42	0.439	0.503
Tb.N, 1/mm	2.20±0.08	2.03±0.07	2.15±0.07	0.635	0.122
Cortical.Th, μm	697±12	688±14	675±12	0.224	0.889
CSMI, mm^4^	7.02±0.15	6.24±0.23	6.47±0.18	0.056	0.037

^1^ Data are presented as the mean±SE (n = 12). BMD–bone mineral density, BV/TV–relative bone volume; Tb.Th–trabecular thickness; Tb.Sp–trabecular separation; Tb.N–trabecular number; Cortical.Th–cortical bone thickness; CSMI–cross-sectional moment of inertia.

## Discussion

This study was designed to evaluate the effects of KYNA on bone structure and mechanical properties in young rats. It is generally recommend that the comprehensive analysis of dietary regulation of bone health should discuss the following aspects: age and sex of used animals, selection of model bone, analysis of bone mineralization, bone mechanical testing, and analysis of trabecular bone. In our study, we used young, rapidly growing rats. As the acquisition of bone mass occurs primarily during childhood and adolescence and more that 90% of adult bone mass is acquired during that periods and improvements of bone mass accumulation following puberty may have positive long-term effects on bone health in adults or older individuals [32, 33], this make neonatal and early postnatal animals an optimal model in study on KYNA regulation of bone health. Our study was performed both on male and female rats, which differs on weight gain rate and female rats typically have lower bone properties compared to male rats given their smaller body size [34]. We used mandible as a model bone, which development and cortical bone mass is minimally influenced by a alterations of body weight-depended mechanical stresses. The analyzed bone traits included assessment of bone mineralization, detailed analysis of the mechanical properties and assessment of microarchitecture of trabecular bone. Conducted analyzes allowed to provide detailed information about the effect of KYNA on bone health.

We found that KYNA chronically administered in a wide range of concentration in drinking water to male and female neonatal rats resulted in the reduced body weight. This effect was observed in all intermediate periods, when rats’ body weight was recorded. Milart et al. [9] recently observed a similar reduction in weight gain in young rats at the weaning exposed postnatally to KYNA in their drinking water at concentration of 25x10^1^ mg/L. In a previous study Turski et al. [35] have reported that long-lasting administration of dietary KYNA did not influence body weight gain and did not reduce lean body mass when administered at a concentration of 25x10^1^ or 25x10^2^ mg/L for 21 days (the same doses as we used in our study). However, Turski et al. performed their study on older female mice (10–12 weeks) and adult male rats with a body weight between 330–390 g, in contrast to our young, female (170–200 g) and male (250–290 g) rats [35]. Other study performed on aged, 22-month-old mice by Isales et al. [36] also shows that KYNA supplementation for 8 weeks has no impact on body weight. In contrast to study by Turski et al. [35] and Isales et al. [36] we started KYNA supplementation in the drinking water immediately after birth, similarly as Milart et al. [9], and continued until postnatal day 60. These results might suggest that KYNA influences body weight gain during the neonatal and early postnatal period of life but not in adulthood. On the other hand, the time when the supplementation should be started to obtain optimal reduction of the weight gain is still not known. Similarly, we cannot exclude, that the restricted body weight gain might be triggered when KYNA supplementation is limited to the post-weaning period. Furthermore, it should be mentioned that reduction of weight gains after KYNA treatment was also observed in adult mice. Agudelo et al. [37] reported that KYNA administered intraperitoneally at the dose of 5 mg/kg of body weight, daily in a single dose for 4 weeks, to adult 4-month-old C57BL/6J mice fed a high-fat diet resulted in reduced their body weight gain and subcutaneous adipose tissue mass, and a reduction in fat mass in mice fed standard diet. In both studies by Agudelo et al. [37] with high-fat diet and Milart et al. [9] the reduction of body weight gain is observed after the same period of KYNA administration.

The deficiency in dietary KYNA could result in dysfunction of adipose tissue, which plays important endocrine functions in regulation of energy homeostasis, insulin sensitivity, lipid and carbohydrate metabolism [11, 37, 38]. It seems necessary to examine in the future research, if this reduction of body weight in our study results from the limitation of lean body mass or the development of white fat tissue.

Body weight gain and adipose tissue interferes with acquisition of bone mass. Alterations in body weight gain are directly associated with the risk of retardation of bone mass gain, as active bone-adipose axis results from a homeostatic feedback system of adipokines, osteoblasts and osteoclasts [39, 40]. We found that a decrease in body weight gain in rats receiving KYNA was not accompanied by a delayed bone growth. Despite the reduction in body mass of young rats following KYNA administration in drinking water in the current study, no effects on densitometric, histomorphometrical parameters or skeletal effects detected on cortical bone were observed in the mandible of the rats receiving KYNA. The only exception is the finding that KYNA, at the lower concentration of 2.5x10^1^ mg/L, slightly increased BMD in males. Interestingly, the BMD of the mandible in the males supplemented with the higher concentration of KYNA 2.5x10^2^ mg/L did not differ from that of the control group. The same result was not observed in the female rats. It should be further investigated, especially as mandible BMD is considered as an indicator of skeletal osteoporosis [41].

Unexpectedly, the biomechanical parameters of the mandibles were affected by KYNA supplementation. Moreover, when a raw bending test data were normalized by body weight and bone geometry (cross-sectional moment of inertia), the positive effects of KYNA supplementation were even more evident. The three-point bending test showed that when male rats were supplemented with KYNA, the increase in bone mechanical endurance was dependent on KYNA concentration, with an increased Young modulus (the bone material equivalent to stiffness) observed in the rats supplemented with 2.5x10^1^ mg/L of KYNA. As Young modulus assesses bone resistance to deformation when loaded, this indicate that their mandible became more rigid, which were also able to withstand higher stress during bending, as it was indicated by the decreased value of elastic energy (the indicator of rigidity) and increased value of ultimate stress (the material indicator of bone strength, representing the ultimate load per bone cross-sectional area) [31, 42]. Thus, when strength was corrected for body weight of the rats (relative ultimate load), it was evident that the mandible became more resistant to deformation or fracture. The above mentioned findings were even more evident in the female rats supplemented with 2.5x10^1^ mg/L of KYNA, in which significantly higher values of relative yield load and elastic stress were observed. The most striking difference, in terms of bone biomechanics, between male and female rats in response to KYNA, is that in female rats the value of work to fracture increased in our study; whereas, in male rats, no difference was seen between treatments. Work to fracture is a integrative measure of a bone’s overall resistance to breakage, depends on the combined values of stiffness, ultimate load, and ultimate strain; it reflects the energy dissipated by the bone structure before failure [42]. In our female rats, the increase of work of fracture results from the increase of ultimate strain observed in females supplemented with KYNA. Thus, the primary difference is that bones get less brittle with KYNA feeding in female rats, but not male rats.

The differences in this postyield behavior may indicate differences in matrix composition or organization of bone organic phase. Bone tissue consists of inorganic (mineral) constituents determining bone density and mechanical strength, while organic components forming matrix contribute mainly to its elastic properties [34]. The organic phase is mostly made of type I collagen, which provides a structural scaffolds to the inorganic phase and contributes to overall bone integrity. There is a study showing that KYNA increases the expression of matrix metalloproteinase MMP1 and alters the production of type I collagen by dermal fibroblasts [43]. However, to date, the possible effect of KYNA on other fibroblast cells has only been suggested [44].

The mandible is both morphologically and functionally different from other bones belonging to the axial skeleton. The mandible is dense with a high proportion of cortical bone. The mandible is considered as a ‘load-bearing’ bone, in which loading during mastication or biting has an impact on its mass, density, and microarchitecture. Nevertheless, from a mechanical point of view, load-bearing bones show some similarities to ‘weight-bearing’ bones, including the long bones of the pelvic limb [30]. For both types of bone, in order to effectively counteract bending and torsion loads and tension or compression, not only is mineralization and the material properties of the bone tissue important, but also the bone geometry and spatial distribution of bone tissue. In contrast to other bones of the axial skeleton the mandible is not directly influenced by gravity or the activity of local muscles, which are anatomically and functionally linked. The general endurance, stiffness and microarchitecture of the trabecular bone making up the mandible are important to counteract mandibular stresses and strains that occur during biting or chewing [30]. For this reason, the mandible is more useful in metabolic or physiological studies [27, 29, 45].

Since KYNA is present in breast milk, baby food formulas and several dietary components [9, 10], we administered KYNA in the most physiological way possible, i.e. through the digestive system. Moreover, as the KYNA was administrated in drinking water, all rats received the same feed and the potential impact of feed texture on mandible morphology and growth was reduced to minimum [46]. It is well known that KYNA given intra-gastrically is absorbed from the gut, distributed to internal organs and tissues, and finally excreted in the urine [35], without permanently elevating serum KYNA levels. This phenomenon could be explained by both the small volume of water consumed by rats via drinking and the rapid excretion of KYNA by the kidneys. The dynamic changes in serum KYNA concentrations, in relation to meal schedules, proposed on the basis of a mathematical model [38] support our explanation.

The kynurenine pathway generates quinolinic acid, an agonist at the N-methyl-d-aspartate (NMDA)-sensitive subpopulation of glutamate receptors, and KYNA, an antagonist of G protein-coupled receptor 35 (GPR35) and broad spectrum glutamate receptors present in osteoblasts and osteoclasts [47]. Glutamate and its precursors are well recognized as important players in the control of bone formation and metabolism. They can modulate bone cell phenotype and result in enhanced bone formation [18, 22, 48–54]. Kynurenine acts through the block of the proliferation of bone marrow mesenchymal stem cells and osteogenic differentiation. Also kynurenine metabolites can modulate cell function and influence bone remodeling through the inhibition of differentiation of osteoblast and the enhancement of RANKL-induced osteoclastogenesis [55, 55, 56]. Another mechanism of modulation bone formation and remodeling of kynurenine pathway involves redox active metabolite, 3-hydroxyanthranilic acid [57]. Clinical observations point to the role of the kynurenine pathway in bone formation and remodelling. Patients with osteoporosis have been shown to exhibit reduced baseline levels of tryptophan compared to healthy controls [58]. However, neither kynurenine nor KYNA content was altered [56]. Similarly, Dinçel et al. [59] observed reduced tryptophan content and unaltered levels of kynurenine in the plasma of humans with osteoporotic hip fractures. Furthermore, reduced levels of erythrocyte tryptophan were found in male idiopathic osteoporotic patients. Noteworthy, in the above mentioned study the bone histomorphometric variables, including wall thickness, trabecular thickness and mineral apposition rate, were positively correlated with tryptophan content in erythrocytes [60]. However, it has been proven that tryptophan metabolism is affected by both gender and age [61].

Kim et al. [62] recently measured the kynurenine content in bone marrow aspirates from humans. Higher kynurenine content was observed in aged subjects with fragility hip fractures, with kynurenine levels inversely associated with bone mass. The authors concluded that increased kynurenine levels during aging may contribute to the bone fragility seen in the elderly through increased bone resorption with a resultant imbalance in bone remodeling [62].

However, there is still lacking clinical trials involving KYNA as a supplement. The reason for this may be the fact of unclear action of KYNA on bone mass and structure, as shown in a recent study on the effect of KYNA administration on femur structure in aged, 22-month-old C57BL/6 mice. In their short communication, Isales et al. [36] have reported the loss in BMD and reductions in trabecular bone histomorphometrical parameters in femur of animals supplemented with high dose of KYNA (375 ppm). These data suggest that in contrast to our young rats, KYNA promotes bone loss in the aged individuals. Thus, the effects of KYNA on bone metabolism may be context dependent and differ during growth and aging. This indicates the need further studies implicated KYNA administration in animal models.

Although, there were several limitations to the present study, the current study also has its advantages. First limitation, no measurements of serum biochemical analyses including KYNA concentrations were performed. This should be included in future studies. Secondly, although two doses of KYNA were used, no clear-cut dose-response was achieved. However, this was the first study showing that KYNA administration did not inhibit bone development in the young rats, as indicated by the lack of changes in histomorphometical, mechanical and material bone parameters. Moreover, X-ray CT scanning allowed us to avoid any mistakes in the calculation of the geometry of the mandible and resulted material properties. Finally, our study included both male and female rats for a subgroup analysis to evaluate the difference between sex in body weight gain and mandibular growth and its biomechanical endurance.

## Conclusions

The results of the current study indicate that KYNA did not disturb bone homeostasis and its implementation to the daily diet is safe in this regard. However, more studies are needed to fully elucidate the role of dietary KYNA in early bone formation and remodeling, especially since it is still unknown whether the effects exerted by KYNA supplementation on mandibular bone are mediated through the actions of kynurenine or tryptophan in the modification of the kynurenine pathway.
